# Addressing Consumer Misconceptions on Antibiotic Use and Resistance in the Context of Sore Throat: Learnings from Social Media Listening

**DOI:** 10.3390/antibiotics12060957

**Published:** 2023-05-24

**Authors:** Sabiha Essack, John Bell, Douglas Burgoyne, Khalid Eljaaly, Wirat Tongrod, Thomas Markham, Adrian Shephard, Elsa López-Pintor

**Affiliations:** 1 Antimicrobial Research Unit, College of Health Sciences, University of KwaZulu-Natal, Durban 4041, South Africa; 2 Graduate School of Health, University of Technology, Sydney, NSW 2007, Australia; john.bell@maroquinhealth.com.au; 3 College of Pharmacy, University of Utah, Salt Lake City, UT 84112, USA; burgoyned@gmail.com; 4 Faculty of Pharmacy, King Abdulaziz University, Jeddah 21589, Saudi Arabia; khalid-eljaaly@live.com; 5 Faculty of Pharmaceutical Sciences, Huachiew Chalermprakiet University, Samut Prakan 10540, Thailand; freshwirat@yahoo.com; 6 Lumanity, London SE1 1PP, UK; thomas.markham@lumanity.com; 7 Reckitt Benckiser Healthcare International Ltd., Slough SL1 3UH, Berkshire, UK; adrian.shephard@reckitt.com; 8 Department of Engineering, Area of Pharmacy and Pharmaceutical Technology, Miguel Hernández University of Elche, 03550 Alicante, Spain; 9 CIBER in Epidemiology and Public Health, 28029 Madrid, Spain

**Keywords:** antibiotics, antimicrobial resistance, antimicrobial stewardship, community pharmacy, COVID-19, misconceptions, upper respiratory tract infection, viral infections, social media listening

## Abstract

A misunderstanding of the mechanism of action and bacterial targets of antibiotics by consumers may drive inappropriate antibiotic use and antimicrobial resistance (AMR). Tackling AMR requires an in-depth understanding of consumer beliefs and misconceptions. We explored consumer conversations on a number of social media platforms on antibiotic use and AMR in the context of sore throat and how coronavirus disease 2019 (COVID-19) affected online conversations between 1 January 2018 and 25 November 2021 across eight countries. Five distinct consumer groups were identified (antibiotic-preserving peer educators, antibiotic-cautious consumers, medication-resistant antibiotic opponents, believers in the strength of antibiotics, determined pro-antibiotic consumers) with a wide spectrum of beliefs around antibiotics in sore throat. Many opinions were based upon misconceptions, the most prominent of which was that antibiotics are strong medications that can treat all types of sore throat. COVID-19 had a multifaceted effect on the sore throat and AMR conversation. Sore throat triggered anxiety as consumers feared it may be a COVID-19 symptom while engagement in conversations around antibiotics for COVID-19 increased. Finally, consumers sought multiple routes to access antibiotics, such as directly from the pharmacy or by attempting to persuade physicians to prescribe. Knowledge obtained from this study could be used to develop focused approaches to dispel consumer misconceptions and mitigate AMR.

## 1. Introduction

Antimicrobial resistance (AMR) is one of the most urgent global health challenges of the 21st century [1] and the World Health Organization (WHO) has consistently listed AMR as one of the top ten global public health threats annually [2]. AMR is also a major source of mortality across the world [3]. In 2019, approximately 4.95 million deaths were associated with bacterial AMR and approximately 1.27 million deaths were directly attributable to AMR globally, positioning it as the third leading cause of death after stroke and ischemic heart disease [4].

Overuse and misuse of antimicrobials significantly contribute to AMR [2,5]. Despite the fact that antibiotics are only indicated for bacterial infections [6,7], common viral illnesses such as the common cold and the flu are frequently treated with antibiotics [6]. For example, a European survey among 26,511 respondents revealed that close to one-third of respondents took antibiotics for a common cold or the flu [6]. Pharyngitis, or sore throat, which is a common manifestation of viral illnesses [8], is also regularly treated with antibiotics. A European survey reported that 13% of respondents took antibiotics for a sore throat [6], while a UK study found that 59% of sore throat consultations resulted in an antibiotic prescription [9].

Inappropriate antibiotic use, whether obtained on prescription or over the counter (OTC), is likely to be influenced by consumer misunderstanding. Studies conducted prior to coronavirus disease 2019 (COVID-19) revealed that up to 71% of patients believed that viruses can be treated with antibiotics [10,11,12,13,14]. Studies conducted during or following the height of the pandemic showed that there was still substantial public misunderstanding, despite widespread public information campaigns around COVID-19 and far greater coverage of topics related to viral infection in the general media. For example, the Sore Throat and Antibiotic Resistance (STAR) study conducted in May 2022 across 12 countries determined behaviors and attitudes related to upper respiratory tract infections, and sought to understand how antibiotics were used for respiratory symptoms [15]. Overall, 55% of respondents (18–64 years) believed that antibiotics kill viruses, while this increased to 60% in 25–34-year-olds [15]. A European survey showed that approximately 40% of respondents incorrectly thought that antibiotics kill viruses and 11% were unsure [6].

It has also been shown that consumers have a poor understanding of AMR. A study using qualitative semi-structured interviews with patients or parents of children presenting with an acute respiratory infection to general practices in Australia, revealed a lack of understanding of what becomes resistant (the person or the bacteria), how patients contribute to AMR, and that resistance can spread between people in close proximity [16]. Furthermore, the STAR study revealed that 36% of consumers were not concerned about AMR [15]. 

Addressing the challenge of AMR as we move past the height of the COVID-19 pandemic requires an in-depth understanding of consumer perceptions and misconceptions around antibiotic use and AMR, so that targeted educational materials/messages and strategies (e.g., consumer engagement models) can be developed and delivered. Social media provides unprecedented communication opportunities between individuals, companies and organizations. In 2021, around one-half of the global population (approximately 4.26 billion people) used social media worldwide [17]. In addition, health organizations commonly use social media as a communication channel to engage their audiences [18]. For example, >80% of state health departments in the United States have social media accounts [19], while the Department of Health in South Africa had more than 100,000 Twitter followers as of March 2020 [20].

The extensive use of social media provides an opportunity to further explore consumer beliefs and conversations regarding antibiotics and AMR and offers opportunities to share appropriate messages through such platforms. The aim of this study was to explore consumer conversations on social media on antibiotic use and AMR in the context of sore throat using Brandwatch, a social listening platform that allows one to monitor and analyze online conversations [21], to determine beliefs and misconceptions and establish how the COVID-19 pandemic influenced online conversations related to sore throat and AMR. Knowledge obtained from this study will inform strategies and resources that can be developed and implemented to address consumer beliefs and misconceptions and help mitigate AMR.

## 2. Results

There were ~760,000 social media posts related to AMR and ~3,500,000 social media posts related to sore throat across the eight countries between 1 January 2018 and 25 November 2021. Table 1 shows the full volume of conversations by topic and country. However, this dataset was then sifted through a human-led qualitative analysis to exclude conversations that fell outside of the topics of interest. Twitter was the dominant platform for advice seeking and experience sharing by people experiencing sore throat, while country-specific healthcare forums where consumers seek advice from peers and healthcare professionals (HCPs) were prominent in some countries. In addition, parenting forums were popular sources where parents and pregnant women with sore throats went for advice about treatment and side effects.

The social media listening exercise revealed three critical findings: (1) distinct consumer groups were involved in conversations on antibiotic use in sore throat with a substantial number of opinions based upon misconceptions; (2) COVID-19 impacted conversations on sore throat and AMR; and (3) COVID-19 impacted how the patient responded to early sore throat symptoms.

### 2.1. Consumer Profiles and Associated Misconceptions around Antibiotic Use in Sore Throat

Five distinct consumer profiles were identified with diverse opinions and beliefs on antibiotic use in sore throat (Table 2).

The first two consumer profiles had the greatest understanding of antibiotics and AMR. Antibiotic-preserving peer educators shared knowledge of the difference between viral and bacterial infections manifesting in sore throat and had a strong opinion on if and when antibiotics should be taken. Consumers in this group tended to share advice only when asked, and while their posts were largely neutral and educational, they appeared to be strongly against antibiotic misuse. Antibiotic-cautious consumers were more hesitant to take antibiotics for a sore throat for different reasons. On one hand, reluctance was due to an awareness of AMR or the knowledge that antibiotics are not indicated for self-limiting sore throat of viral etiology. On the other hand, reluctance stemmed from the perception that antibiotics are ‘too strong’ a medication to take for something as trivial as sore throat; the tone of the conversation by these consumers made it seem that they had a moral reluctance to take antibiotics for sore throat (e.g., that it felt wrong or unethical for them to do so). These consumers often preferred home remedies and symptom relief; however, this was not driven by a belief that ‘natural’ solutions are superior, but rather by the knowledge that sore throat is self-limiting and will resolve over time with symptomatic management.

The third group were categorized as medication-resistant antibiotic opponents, who appeared to prefer home remedies and were reluctant to take antibiotics due to a misunderstanding of AMR. Consumers in this group believed that AMR could be ‘caught through antibiotics’ and believed that antibiotics make bacteria in their body stronger, which was partially reinforced by a concern about missing antibiotic doses and forgetting to finish the full course of treatment. This group also included pregnant women and parents who were hesitant to give/take antibiotics because of a perception that they could cause side effects or weaken the immune system of their child.

The final two consumer profiles appeared to be supporters of antibiotics with a lesser understanding of the mechanism of action of antibiotics. Consumers that were believers in the strength of antibiotics were typically keen to use antibiotics as they believed they are ‘strong medications’ that provide quick relief of symptoms. Consumers in this group were unable to make the distinction between viral and bacterial infections and often felt frustrated when their sore throat did not clear up with antibiotic treatment. Determined pro-antibiotic consumers were part of a smaller group. They often shared their beliefs that antibiotics are ‘strong medications’ and proactively attempted to source antibiotics by trying to persuade their physician. Some consumers even went directly to a pharmacy to obtain antibiotics OTC.

A number of consumer misconceptions were identified, with the most prevalent often being misinterpretations of the science behind AMR rather than outright fallacies. The most common consumer misconception was that antibiotics are strong medications that can treat all types of sore throat (Table 3).

### 2.2. Impact of COVID-19 on the Sore Throat AMR Conversation

COVID-19 had a multifaceted effect on sore throat and AMR conversations. Firstly, sore throat triggered anxiety in consumers as they feared it may be a symptom of COVID-19, which prompted consumers to test for COVID-19. Secondly, the pandemic led to consumers engaging in conversations around antibiotics and the distinction between viral and bacterial infections in the context of COVID-19, and antibiotics not being effective against viral infections. Furthermore, early in the pandemic, commentary by consumers and HCPs was directed towards people being prescribed antibiotics for COVID-19. A minority of consumers suggested that antibiotic prescriptions for COVID-19 may play a role in the prevention of opportunistic bacterial infections. Finally, public interest in natural remedies for sore throat remained prominent, and possibly even increased during the pandemic, driven by a reluctance to visit healthcare centers for fear of contracting COVID-19 or overburdening the health system, as well as a desire to lead a healthy lifestyle focused on wellness.

In addition to consumers, the study found that other stakeholders played a larger part in conversations that included sore throat, AMR and COVID-19. For example, activities carried out during World Antibiotic Awareness Week (WAAW) by government agencies, health authorities, local branches of the WHO and regional healthcare bodies focused on raising awareness of AMR and the dangers it poses in general, and garnered significant coverage through online platforms. A small proportion of individual campaigners and consumers (largely made up of individuals who fit into peer educator and antibiotic-cautious consumer profile) joined the conversations. Supportive campaigns and conversations around AMR became less prominent during the height of the COVID-19 pandemic, as health authorities focused on tackling COVID-19. Furthermore, many stakeholders outside of government influenced the AMR conversation, including peer educators (e.g., practicing or former HCPs who have carved out a platform in social or traditional media and who publish their own content aimed at combatting antibiotic overuse) and non-healthcare figures in the public domain (e.g., models, TV presenters and politicians).

### 2.3. Impact of COVID-19 on Patient Response to Early Sore Throat Symptoms

Consumers sought advice for sore throat relief through multiple routes. Consumers seeking advice from people in an individual’s social network was common in online sore throat conversation. Some consumers frequently visited the pharmacy for OTC symptom relief if they did not already have this at home. Meanwhile, other consumers visited their physician or pharmacist immediately, especially when they were unsure if their symptoms were COVID-19-related. In some markets (Russian Federation, Romania, Brazil and Mexico), consumers with sore throat were more likely to demand specific antibiotics from their physician. In other markets (Germany and Italy), consumers attempted to persuade their physician to prescribe antibiotics by stating that they were necessary or exaggerating their symptoms. Antibiotics or antibiotic-containing products frequently mentioned by people experiencing sore throat included amoxicillin (Thailand), amoxicillin combined with clavulanic acid (Thailand), penicillin V (Germany), Benzetacil (benzyl penicillin) (Brazil), Grammidin (gramicidin) (Russian Federation), and Bioparox (fusafungine) spray (Romania). A trigger for visiting primary care/family physicians was when symptoms worsened or lingered for some time and depended on factors related to COVID-19, such as the desire to test and fear of infection, as well as factors related to the health systems (e.g., cost of services). In addition, in countries where antibiotics were more commonly available without a prescription directly from the pharmacy (even in situations where it is illegal), some consumers went directly to the pharmacy to access treatment.

## 3. Discussion

To our knowledge this is the first study to examine consumer beliefs and conversations on sore throat, antibiotics and AMR, and the impact that the COVID-19 pandemic has had on such conversations, using posts from multiple social platforms and forums. Our study assessed a large number of social media posts, which allowed us to unpack the diverse consumer beliefs related to antibiotic use. We found that consumer conservations were clustered, which allowed us to define five distinct groups, with many groups’ opinions based upon misconceptions. In addition, COVID-19 impacted conversations around sore throat and AMR and how patients respond to early sore throat symptoms.

Beliefs and misconceptions observed among the five groups aligned with previous studies. At one end of the scale were antibiotic-preserving peer educators and antibiotic-cautious consumers who had the greatest understanding of antibiotics and AMR. Other survey-based studies also found that there were a proportion of patients (between 36.5% and ~50%) who clearly understand that antibiotics are only effective against bacterial infections [6,11,13]. Previous studies have also revealed an understanding of AMR among patients. A large proportion of patients (73.7% to 85%) were aware that unnecessary use of antibiotics compromise their efficacy in the future [6,12,13], while 72% were aware that many infections are becoming increasingly resistant to treatment by antibiotics [10]. Furthermore, 84.1% of patients understood that bacteria can become resistant to antibiotics, while 61.5% understood that the prescribed course of antibiotics needed to be taken or they may not work in the future [13]. Meanwhile, determined pro-antibiotic consumers and believers in the strength of antibiotics who were in favor of antibiotics had the least understanding of how antibiotics work, believing that antibiotics are strong medications suitable for all types of sore throat (bacterial and viral). Survey-based studies have revealed that a substantial number of patients (36.5% to 71%) believe that antibiotics are effective in treating viral infections [6,10,11,12,13,14,15]. We also found that determined pro-antibiotic consumers proactively attempted to source antibiotics by trying to persuade their physician. Similarly, a study that utilized video recordings of adult primary care consultations found that many patients conveyed subtle forms of pressure towards their physician to obtain antibiotics [22]. Sitting between consumers with the greatest understanding of antibiotics and AMR and those in favor of antibiotics in our study were medication-resistant antibiotic opponents, who were reluctant to take antibiotics due to a misunderstanding of AMR. A questionnaire-based study also found that there was a reluctance among some patients to take antibiotics for reasons different to what was found in our study. In the questionnaire-based study, patients were reluctant to take antibiotics due to a concern about bacterial resistance, although it is important to note that patients who expressed this concern had experienced challenges with antibiotic treatment, thought that antibiotics were generally ineffective and did not hesitate to stop treatment inappropriately [23]. The belief that antibiotics are strong medications, capable of treating bacterial and viral infections was evident across all eight countries included in the study.

For HCPs to help address consumer misconceptions, we recommend a multifaceted approach. Firstly, we would suggest that HCPs become knowledgeable on the identified consumer profiles and the desired messages that should be conveyed to bring a shift in belief/behavior. Secondly, as it may be difficult to determine the beliefs of a consumer on first contact, we would suggest utilizing open-ended statements and questions to explore what consumers are looking for and identify any misconceptions. Once this information has been gathered, messages can be customized and relevant decision aids and knowledge bites on appropriate antibiotic use and symptomatic management can be provided, to address misconceptions and empower the consumer to make appropriate health decisions (Table 4). A simple call-to-action model, such as the ACE model for engagement developed by the Global Respiratory Infection Partnership, would be useful to ensure that HCPs are clear on the steps that should be taken to bring about a belief/behavior change. The ACE model recommends that HCPs: **A**sk questions to establish the understanding of AMR by the consumer and identify misconceptions; **C**ustomize messages according to the consumer profile; **E**mpower consumers with the knowledge to understand the cause of their illness and why symptomatic relief can be effective.

The large number of social media posts analyzed in our study indicated that social media platforms are regularly used by various stakeholders to share opinions and perspectives and engage in discussions. Other studies have also found that social media is widely used by patients to source information and connect with their peers, healthcare organizations and HCPs [19,24,25,26]. The extensive use of social media provides opportunities to leverage such platforms to provide targeted education/messaging on public health issues such as AMR in a swift manner, which could help dispel misconceptions and translate into remedial action. When thinking about developing targeted education and messaging for use on social media, we would recommend the consideration of multiple factors. Firstly, it is important to consider the social platform that is used, whether there are any guidelines that should be considered, and if the message will translate as intended. For example, Twitter, which was the most commonly used platform in our study, has a maximum character limit of 280 [27]. Secondly, the format of social media content should be considered. By providing succinct content that is straightforward and visually engaging, the consumers’ attention can be captured and sustained. It is also critical to use language that consumers will understand versus more scientific language. Based on the number of misconceptions observed in our study, it could be argued that language and messaging on antibiotics and AMR may need to change. Furthermore, it is important that targeted education and messages are tailored according to the different profiles, to ensure that the appropriate misconceptions are being dispelled. Finally, the age group of the target audience should be considered and social media platforms that are more commonly used by these audiences should be utilized.

Along with the development of social media content to provide education and convey messages, consumers and HCPs that are less familiar with social media should not be overlooked. We recommend that targeted educational materials (e.g., short videos, easy-to-read booklets) remain available within the healthcare setting to ensure all consumers continue to receive relevant education. Notably, future HCPs (including community pharmacists and pharmacy counter assistants) are likely to be digitally native in their forms of communication and digital social engagement, meaning that they may not need as much training and education in this area. However, it is essential that future HCPs receive appropriate training on the different consumer profiles that have been identified in this study, along with AMR and antimicrobial stewardship [28], so that they can convey the correct information effectively to consumers, both in person and via social media.

Social media can be a useful tool for health promotion, providing communication opportunities between several stakeholders and audiences [29]. However, it can also be a source for misinformation [30]. Our study did not examine where consumer misconceptions originated from; however, social media could have played a part. We recommend that various stakeholders play a role in helping to mitigate the spread of misinformation and convey the correct messaging to consumers. Some stakeholders may need to work with social influencers, who are becoming more common in the healthcare space, as they have a greater understanding of the social media space and better reach. It is important to note that if stakeholders utilize influencers, they will not have the same control on content; therefore, it is important to educate influencers on the objective and the evidence that underpins the intended messaging and consider legal/regulatory frameworks.

COVID-19 had a multifaceted effect on sore throat and AMR conversations. For example, sore throat triggered anxiety in consumers as they feared it may be a symptom of COVID-19, which prompted them to carry out diagnostic testing. Interestingly, supportive campaigns and conversations around AMR became less prominent during the height of the COVID-19 pandemic, as health authorities focused on tackling COVID-19. Given that AMR remains a major health issue, we recommend that communications should be refocused on how inappropriate antibiotic use can contribute to AMR and the threat that it poses and should leverage our learnings from the COVID-19 pandemic.

In addition to targeted materials, messages and approaches to address consumer misconceptions on sore throat and antibiotic use, additional strategies could be employed to further help in the fight against AMR. Most cases of sore throat (up to 80%) are of viral etiology, meaning that antibiotics will not work [31]. Furthermore, as the symptoms of bacterial and viral infections are similar, it can be difficult to make a diagnosis from symptoms alone [32]. Therefore, point-of-care testing (POCT) could be implemented in primary care settings (e.g., pharmacy and general practice) to help distinguish between the cause of sore throat (viral or bacterial) in a rapid manner, enhance the quality of antibiotic prescribing decisions and help to guide self-care options for consumers [32]. Although there are challenges associated with POCT, such as the training required for HCPs, throat swab testing has become more familiar for patients following the COVID-19 pandemic. In addition, previous studies have demonstrated that POCT within the community pharmacy setting can reduce the number of inappropriate antibiotic prescriptions [33,34]. Bacterial microorganisms are capable of developing resistance to some antimicrobial agents through various mechanisms of resistance [3]. Most cases of sore throat with a bacterial etiology are caused by *Streptococcus pyogenes* (*S. pyogenes*) [35]. Penicillin resistance has not been reported for *S. pyogenes*; therefore, it is considered the antibiotic of choice for the treatment of group A streptococcal pharyngitis [36]. However, in cases where treatment fails (for example due to inadequate dosing), it is important to detect potential drug resistance and confirm susceptibility of the bacterial strain, to ensure that the most appropriate alternative antibiotic is prescribed [36,37,38]. Beyond POCT, antimicrobial susceptibility testing (AST) could be utilized to determine which antimicrobials will inhibit bacterial growth and ensure that the correct antibiotic regimen is provided to patients [37]. Various AST methods are available, which can provide accurate detection of antimicrobial resistance mechanisms when used correctly. However, it is important to note that they do come with some challenges, such as the speed in which results are produced from microbial culture and susceptibility testing; it is a two to three day process, which could in turn delay antibiotic administration [38,39]. It is important to also note the study limitations. Firstly, the study relied on qualitative rather than quantitative analysis. Therefore, future studies that assess the prevalence of different antibiotic consumer profiles and quantitatively characterize conversations across different social media platforms would be beneficial. In addition, as a qualitative approach was taken and the analysis looked at the totality of conversations within a defined timeframe, there was no ability to define a sample size. Secondly, the demographics present online in the discussion of antibiotics and sore throat may not be representative of the population. Thirdly, Brandwatch only has access to public social media accounts, meaning that there could have been significant conversations among people experiencing sore throat on private social media channels (e.g., individuals’ private Facebook accounts) that were not captured. Finally, it is important to note that retweets/reposts formed part of the overall dataset, and it is possible that automated posts/bot accounts could have formed part of the dataset. However, as analyzers focused on conversations by individuals first and foremost in the qualitative analysis, we do not expect retweets/reposts or bot accounts to have had a significant impact on our findings.

## 4. Materials and Methods

**Figure 1:**
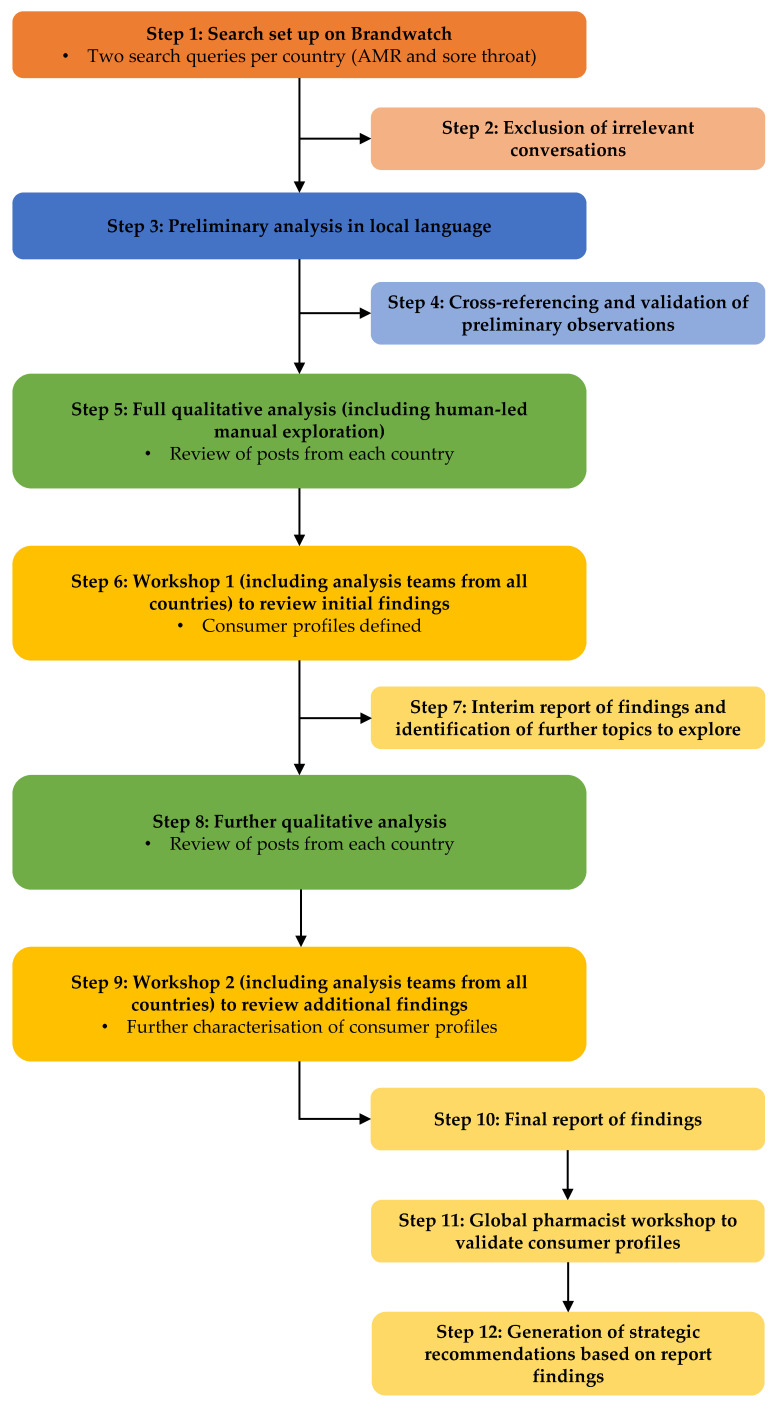
Schematic of study methodology. AMR, antimicrobial resistance.

This was a multinational, observational, consumer content analysis study initiated and commissioned by Reckitt, the maker of Strepsils. The study was conducted across eight countries: Germany, Italy, Spain, Mexico, Brazil, Thailand, Romania and the Russian Federation. Social media searches were conducted between 1 January 2018 and 25 November 2021 to explore conversations around AMR and sore throat and the effect of COVID-19 on online conversations by contrasting the conversation before and after 1 January 2020. The master search terms in English are provided in Table 5 and Table 6. These terms were translated as appropriate. Social media posts from Twitter, Reddit, YouTube, forums, blogs, Facebook and Instagram posted by the general public, patients (people experiencing sore throat), healthcare organizations and HCPs in the main local language were included. Figure 1 provides an overview of the steps involved in the study.

During Step 1, Brandwatch, a social listening platform that allows one to monitor and analyze what is being said online about brands, people and products [21], was utilized to set up two separate search queries that were translated into local language by individual analysts. The search queries captured relevant conversations across social media platforms around AMR (explicit and implicit references to this) and sore throat, and among the abovementioned stakeholders, which was followed by data cleaning to remove irrelevant conversations as Step 2. This iterative process involved the review of a portion of the dataset to identify any recurring unintended themes (e.g., the term ‘multidrug resistant’ pulled in many cancer-related conversations) and subsequent adjustment and refinement of the search terms. In addition, any records that were not geotagged to one of the eight countries of interest were removed from the data set at this stage.

Step 3 consisted of a preliminary analysis, which was conducted in local languages. During this step, social media conversations were reviewed by analysts to identify and summarize the most prevalent themes (‘conversation drivers’) of the conversations around antibiotics in the context of sore throat. Data were then distilled into an initial update by cross-referencing and validating the preliminary observations (Step 4).

The first qualitative analysis was conducted (Step 5) by a team of social media analysts who were proficient in the local language of each country. The analysis explored how antibiotics were being perceived and used by consumers within the context of sore throat. Data collection via Brandwatch was supplemented with extensive human-led manual identification and exploration of additional forums and data-limited channels. These additional country-specific sources were identified by analysts with local country knowledge, and through internet searches using the terms that had featured most prominently within the conversations identified via Brandwatch. The full qualitative analysis included the review of ~2500 posts per country and focused on the following topics: sore throat diagnosis; sore throat tests; sore throat self-treatment; sore throat hospital treatment; sore throat treatment with antibiotics; health literacy; AMR in the context of sore throat; consumer perception of antibiotic policy (in the context of the country stance on antibiotic use) and campaigns; antibiotic availability; key influencers (celebrities and public figures) discussing AMR; and the biggest changes in AMR conversations before and after the advent of the COVID-19 pandemic.

Following qualitative analysis, the first workshop was held to define the consumer profiles (Step 6). Workshop participants included analysis teams from all countries, Reckitt and Lumanity (the agency commissioned to carry out the study). Key beliefs and perceptions were summarized and organized along two axes: ‘in favor of antibiotics’ versus ‘opposed to antibiotics’ and ‘health literate’ versus ‘misinformed’. Where multiple individual beliefs were clustered together, these were grouped into different consumer profiles. An interim report was generated outlining the preliminary findings and identifying the additional key questions to explore (Step 7).

Step 8 consisted of a subsequent qualitative analysis to further refine and add detail to the consumer profiles, including discussions centering on consumer perceptions of interactions with HCPs in the path of receiving antibiotics; biggest changes in the sore throat conversations before and after the advent of the COVID-19 pandemic; sore throat channel breakdown analysis to determine which social media channels were used by different stakeholder groups and which were the most prominent types of conversation on these channels; WAAW; and Strepsils. During the second workshop (Step 9), analysis teams from each country, Reckitt and Lumanity assessed the prevalence of consumer profiles and shifts due to COVID-19. Key findings from the qualitative analyses and the workshops were distilled into a final report (Step 10) detailing the main areas of implications and the key messages from the study.

Key themes from the social media research were presented to a global group of pharmacists attending a Global Respiratory Infection Partnership workshop, which was held at an international pharmacy conference (80th International Pharmaceutical Federation (FIP) World Congress of Pharmacy and Pharmaceutical Sciences 2022). The workshop assessed whether consumer profiles and perceptions were recognized by pharmacists in their country. In addition, the workshop aimed to define the questions consumers in each profile should be asked and potential responses that could be provided to address misconceptions (Step 11). The analysis formed the basis for our recommended strategies and resources that could be implemented to address consumer misconceptions and help tackle AMR (Step 12).

## 5. Conclusions

Our social media listening study illustrated a number of misconceptions on sore throat, antibiotic use and AMR, and further showed that COVID-19 has impacted conversations and the patient response to early sore throat symptoms. The knowledge obtained from the study provides an opportunity to address the diverse consumer misconceptions through HCP interventions, both in person and in the social media space, and via other stakeholders, to effectively mitigate the challenge of AMR. One of the objectives of the WHO global action plan on AMR is to increase the awareness and understanding of AMR using strategies that focus on communication. Therefore, future efforts should focus on developing materials and messages that tackle identified misconceptions in a succinct and impactful way. In addition, finding the right people and channels to spread correct messaging to address misconceptions is of great importance.

## Figures and Tables

**Table 1:** Volume of conversations analyzed in the social listening exercise.

Country	Number of AMR Conversations	Number of Sore Throat Conversations
Germany	125,000	447,000
Italy	196,000	339,000
Spain and Mexico	168,000	494,000
Romania	5000	20,000
Russia	102,000	816,000
Brazil	93,000	1,400,000
Thailand	73,000	Data unavailable

**Table 2:** Consumer profiles deduced in the social media listening exercise and key beliefs observed for each profile.

Consumer Profiles				
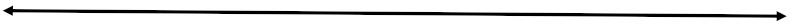				
Opposed to Antibiotics				In Favor of Antibiotics
Antibiotic-Preserving Peer Educators	Antibiotic-Cautious Consumers	Medication-Resistant Antibiotic Opponents	Believers in the Strength of Antibiotics	Determined Pro-Antibiotic Consumers
Antibiotic-preserving peer educators feel it is their duty to educate friends, family and acquaintances about the importance of following antibiotic prescriptions correctly, and of mitigating antibiotic resistance	Antibiotic-cautious consumers believe antibiotics are unnecessary for sore throat, driven by either explicit awareness of AMR or an implicit reluctance to take ‘hard’ medicine	Medication-resistant antibiotic opponents are opposed to antibiotics because of a reluctance towards taking medication, a preference for natural/holistic treatment, and even outright conspiracy in some instances	Believers in the strength of antibiotics are characterized by the opinion that antibiotics are a ‘strong’ medication, and they fundamentally do not make the viral/bacterial distinction	Determined pro-antibiotic consumers are convinced that their sore throat warrants antibiotic treatment and that, as a ‘strong medicine’, it will enable them to recover more quickly
**Key Beliefs ^1^**				
“I need to educate my peers around how to use antibiotics appropriately”	“I don’t want antibiotics because they are unnecessary/this is contributing to AMR”	“I would always prefer natural/holistic treatments to antibiotics”	“I want antibiotics because they are a strong medication and I want to get better quickly”	“I should be prescribed antibiotics, but my doctor won’t give them to me”
“You should only take antibiotics if you have white plaques/a fever for more than three days/if your child also has ear pain or cough”	“I am annoyed because my doctor has prescribed me antibiotics unnecessarily”	“I don’t want to ‘catch’ AMR/I don’t want to make the bacteria in my system stronger”	”Because my sore throat is getting worse, I am going to seek out and take antibiotics”	“I need antibiotics and have therefore got them directly from a pharmacy”
“Antibiotics should be prescribed by doctors and prescriptions should be followed thoroughly”	“Taking antibiotics for sore throat is dangerous and stupid”	“I don’t want antibiotics because they are harmful to my/my child’s immune system/they can cause side effects”	“I am dissatisfied about why antibiotics aren’t working for my sore throat, I thought they were a powerful medication”	“Of course, luckily enough we live in Romania where you can get antibiotics without a prescription”
**Prevalence of Consumer Profiles**				
▲DE, ES, BR, TH, IT; ▼RU, RO	▲DE, RU	▲RU	Prevalent in all markets	▲ES, RU, RO, TH, BR, MX; ▼DE, IT

AMR, antimicrobial resistance; BR, Brazil; DE, Germany; ES, Spain; IT, Italy; MX, Mexico; RO, Romania; RU; Russian Federation; TH, Thailand. ^1^ Key beliefs are quotes developed by analyst teams to illustrate/summarize the beliefs being expressed; ▲Higher prevalence of consumer profiles in markets indicated; ▼Lower prevalence of consumer profiles in markets indicated.

**Table 3:** Top four prevalent consumer misconceptions identified across the consumer profiles.

Rank	Consumer Belief
1	People experiencing sore throat often alluded to the belief that antibiotics are strong medications, which are effective at treating all types of sore throat They do not make the distinction between viral and bacterial infections Antibiotics are perceived to be more effective at resolving sore throat compared with pharmacologic symptomatic relief or home remedies
2	Some people experiencing sore throat express the belief that antibiotics will be able to help them recover from sore throat more quickly when compared with pharmacologic symptomatic relief or home remedies They believe this to be the case for all types of sore throat, and do not make the distinction between viral and bacterial infections
3	Some people experiencing sore throat discussed their fear that if they take too many antibiotics, or do not take them properly, they will ‘catch AMR’ or will personally become resistant to antibiotics
4	People experiencing sore throat also mention the belief that if they take too many antibiotics, or don’t take them properly, they will make the bacteria in their system stronger or create a ‘superbug’ in their body which will only affect them, rather than having an impact on the community

AMR, antimicrobial resistance.

**Table 4:** Strategies to address misconceptions related to antibiotic strength and AMR.

	Misconception	Belief/Behavior Shift Required	Strategies to Address
**Misconceptions about antibiotic strength**	“Antibiotics will help me to get better more quickly” “Antibiotics are strong medications which are more effective at treating my sore throat [regardless of whether it is viral or bacterial]”	Antibiotics are only effective for bacterial infections; their primary role is not to alleviate symptoms, but to kill bacteria Antibiotics are ineffective for viral infections—and therefore for most sore throat conditions [that are viral] Effective OTC products are available to manage the painful symptoms quickly, without the need for antibiotics	**1. ASK questions to understand where the patient is in their AMR journey and identify misconceptions they may have** Ask questions about the patient’s symptoms Explore what benefit the patient is looking for beyond antibiotics (e.g., pain relief) **2. CUSTOMIZE your messages to address patients’ concerns using simple yet evidence-driven messages** Leverage patient decision aids and easy-to-use materials to explain the notion of appropriate antibiotic use Create short videos and engage with influencers to increase awareness through social media **3. EMPOWER patients with the knowledge needed to understand the cause of their illness and why evidence-based symptomatic relief can be effective for URTIs** Explain the difference between viral and bacterial infections and that the symptoms will not resolve with antibiotics if the infection is viral. State that most sore throats are of viral etiology Advise on alternatives to antibiotics that offer relief of patient’s specific symptoms
**Misconceptions about antimicrobial resistance**	“I’ve never used antibiotics so I can’t have resistant infections” “If I take too many antibiotics or don’t take them properly, I will ‘catch AMR’ or I will personally become resistant to antibiotics”	AMR is a public health issue and not only a personal issue Inappropriate use of antibiotics can lead to bacteria becoming resistant to antibiotics	**1.** **ASK questions to understand where the patient is in their AMR journey and identify misconceptions they may have** Ask questions to establish patient understanding and explore the route of misconceptions around AMR **2. CUSTOMIZE your messages to address patients’ concerns using simple yet evidence-driven messages** Use plain language to educate patients around the importance of preserving antibiotics for when we need them the most and the consequences of inappropriate antibiotic use Bring to life why this might matter to the person in front of you (e.g., affecting the effectiveness of antibiotics for future operations) **3. EMPOWER patients with the knowledge needed to understand the cause of their illness and why evidence-based symptomatic relief can be effective for URTIs** Leverage patient decision aids and easy-to-read materials to explain appropriate antibiotic use and evidence-based symptomatic relief alternatives

AMR, antimicrobial resistance; OTC, over the counter; URTI, upper respiratory tract infection.

**Table 5:** Master English search terms for ‘Sore Throat’ used on Brandwatch.

Standalone Terms	Terminology Associated with Sore Throat	Anchored Terms of Descriptions of Sore Throat	Anchored Terms for Treatment	Anchored Terms: Conditions where Sore Throat Is a Symptom
“sore throat” OR sorethroat	“streptococcal infection”	*anchor term:* **throat NEAR/3**	*anchor term:* **throat NEAR/5**	*anchor term:* **throat NEAR/5**
*“soar throat”* OR *soarthroat*	streptococcus	scratchy	Halls	“acid reflux”
“strep throat”	pharyngitis	dry	Riccola	COVID-19
“throat pain”	laryngitis	irritated	Strepsils	COVID
“swollen glands”	tonsillitis OR *tonsilitis*	allergies	lozenge	Coronavirus
“throat relief”		“lost voice”	ibuprofen	flu *
(lost OR lose OR losing) NEAR/1 voice		hoarseness	paracetamol	influenza
“Centor score” OR “Centor test” OR “Centor criteria”		swell * OR swollen	nurofen	virus
“FEVERPain” OR (FEVER NEAR/1 Pain)		hurt *	penicillin	“viral infection”
“McIsaac score” OR “McIsaac test”		pain *	amoxicillin	“coronavirus symptoms”
“strep test” OR “strep A test”		sore OR *soar*	Amoxil	“COVID symptoms”
“throat swab”		itchy	cephalexin	“COVID-19 symptoms”
throat NEAR/3 (“CRP test” OR “CRP level” OR “C-reactive protein”)		inflam *	Keflex	#COVID_19
		discomfort	cefadroxil	#COVID-19
		uncomfortable	clindamycin	#COVID
		“fxxxxd up” OR “is fxxxxd”	azithromycin	allergies
		“killing me”	clarithromycin	allergy
		“bothering me”	cephalosporin	mononucleosis
		“can’t eat” OR “cannot eat” OR “difficulty eating”	tertracycline	mono
		“can’t drink” OR “cannot drink” OR “difficulty drinking”	macrolide	mumps
			fluoroquinolone	“gastroesophageal reflex disease”
			glycopeptide	GERD
			Mebucaina	streptococcal pharyngitis
			tyrothricin OR Mybacin OR Anginovag	croup OR laryngotracheitis OR laryngotracheobronchitis
			gramicidin OR grammicidin	sinusitis OR “sinus infection”
			lemocin	rhinitis OR rhinosinusitis
			dorithricin	“common cold” OR “a cold”
			antibiotic * OR “anti B” OR “anti-B” OR “anti-B’s” OR “anti Bs”	infection OR infected
			carbapenem	
			methicillin	
			vancomycin	

Notes for reading the table: quotation marks indicate these terms were searched for as an exact phrase, as they appear in a post; terms preceded by the hash symbol (#) are hashtags that were used to identify posts on a specific topic on Twitter; anchor terms were used to only capture posts containing both the anchor term and the search term; terms in italics are common misspellings; NEAR/5 results when the search term appears five words or less away (either side) from any of the preceding terms; terms ending in (*) indicate the use of the wildcard operator to include alternative suffixes, allowing the capture of variant spellings, conjugations, plurals, etc. (e.g., searching for swell* also captures results including swells, swelled, swelling).

**Table 6:** Master English search terms for ‘AMR’ used on Brandwatch.

Condition Terms	“Superbug“ Infection Terminology	Antiviral/Antifungal Resistance Terminology	Anchored Terms	Exclusions
“antimicrobial resistance”	“superbug”	*anchor terms:* (antiviral OR “anti-viral” OR antifungal OR “anti-fungal” OR Gocovri OR Symadine OR Symmetrel OR amantadine OR Flumavine OR rimantadine OR Tamiflu OR oseltamivir OR Relenza OR zanamivir) NEAR/3	*anchor terms:* (antibiotic * OR “anti B” OR “anti-B” OR “anti-B’s” OR “anti Bs” OR penicillin OR amoxicillin OR Amoxil OR cephalexin OR Keflex OR cefadroxil OR clindamycin OR azithromycin OR clarithromycin OR cephalosporin * OR tertracycline * OR macrolide * OR fluoroquinolone * OR glycopeptide * OR tyrothricin OR Mybacin OR Anginovag OR gramicidin OR grammicidin OR lemocin OR dorithricin OR carbapenem OR methicillin OR vancomycin) NEAR/3	“fossil fuel”
“antibiotic resistance”	“methicillin-resistant *Staphylococcus aureus*”	overuse * OR overprescribe * OR overprescription	“do not respond” OR “does not respond”	agriculture
“antibiotic addiction”	MRSA	“stop working” OR “stops working” OR “stopped working”	response	farming
“antibiotic resistant pathogens”	“Carbapenem-resistant Enterobacteriaceae”	“doesn’t work” OR “does not work”	resilient	animals
“multidrug-resistant bacterium”	CRE		resilience	climate
AMR	“ESBL-producing Enterobacteriaceae”		“biothreat pathogens”	food
“drug resistant”	“ESBL bacteria”		“health threat”	“food safety”
“drug resistance”	“Vancomycin-resistant *Enterococcus*”		#onehealth	“food science”
“when antibiotics fail”	VRE		#AntibioticStewardship	livestock
“antibiotics overuse”	“Multidrug-resistant *Pseudomonas aeruginosa*”		abuse	“animal health”
“World Antimicrobial Awareness Week”	MDR		resistance	“animal welfare”
#WAAW	“Multidrug-resistant Acinetobacter”		virus	environment
#AMR	“H30-Rx”		viral	environmental
#AntimicrobialResistance	*E. coli* H30-Rx		coronavirus	“AMR Industry Alliance”
superbug			flu	“American Medical Response”
#superbug			“the cold”	“Aston Martin Racing”
#AntibioticResistantInfections			“a cold”	“AMR Consultancy”
#EAAD			#ECDC	“European Alliance Against Depression”
			“European Centre for Disease Prevention and Control”	pig * OR cow * OR cattle *
			overuse * OR overprescribe * OR overprescription	
			“stop working” OR “stops working” OR “stopped working”	
			“doesn’t work” OR “does not work”	

Notes for reading the table: quotation marks indicate these terms will be searched for as an exact phrase, as they appear in a post; terms preceded by the hash symbol (#) are hashtags which are used to identify posts on a specific topic on Twitter; anchor terms are used to only capture posts containing both the anchor term and the search term; terms in italics are common misspellings; ~6 returns results when the search term appears six words or less away (either side) from any of the preceding terms; terms ending in (*) indicate the use of the wildcard operator to include alternative suffixes, allowing the capture of variant spellings, conjugations, plurals, etc.

## Data Availability

The data are contained in the article.
